# Adaptive statistical iterative reconstruction (ASIR) affects CT radiomics quantification in primary colorectal cancer

**DOI:** 10.1007/s00330-019-06073-3

**Published:** 2019-03-18

**Authors:** Davide Prezzi, Katarzyna Owczarczyk, Paul Bassett, Muhammad Siddique, David J. Breen, Gary J. R. Cook, Vicky Goh

**Affiliations:** 1 School of Biomedical Engineering and Imaging Sciences, King’s College London, King’s Health Partners, Lambeth Wing, St Thomas’ Hospital, London, SE1 7EH UK; 2 Department of Radiology, Guy’s and St Thomas’ NHS Foundation Trust, Lambeth Wing, St Thomas’ Hospital, London, SE1 7EH UK; 3 Department of Clinical Oncology, Guy’s and St Thomas’ NHS Foundation Trust, Lambeth Wing, St Thomas’ Hospital, London, SE1 7EH UK; 4 Statsconsultancy Ltd., 40 Longwood Lane, Amersham, Bucks HP7 9EN UK; 5 University Hospital Southampton NHS Foundation Trust, Tremona Road, Southampton, SO16 6YD UK; 6 King’s College London & Guy’s and St Thomas’ PET Centre, Lambeth Wing, St Thomas’ Hospital, London, SE1 7EH UK

**Keywords:** Multidetector computed tomography, Image processing, Computer-assisted, Colorectal neoplasms, Fractals

## Abstract

**Objectives:**

To investigate whether adaptive statistical iterative reconstruction (ASIR), a hybrid iterative CT image reconstruction algorithm, affects radiomics feature quantification in primary colorectal cancer compared to filtered back projection. Additionally, to establish whether radiomics from single-slice analysis undergo greater change than those from multi-slice analysis.

**Methods:**

Following review board approval, contrast-enhanced CT studies from 32 prospective primary colorectal cancer patients were reconstructed with 20% ASIR level increments, from 0 to 100%. Radiomics analysis was applied to single-slice and multi-slice regions of interest outlining the tumour: 70 features, including statistical (first-, second- and high-order) and fractal radiomics, were generated per dataset. The effect of ASIR was calculated by means of multilevel linear regression.

**Results:**

Twenty-eight CT datasets were suitable for analysis. Incremental ASIR levels determined a significant change (*p* < 0.001) in most statistical radiomics features, best described by a simple linear relationship. First-order statistical features, including mean, standard deviation, skewness, kurtosis, energy and entropy, underwent a relatively small change in both single-slice and multi-slice analysis (median standardised effect size *B* = 0.08). Second-order statistical features, including grey-level co-occurrence and difference matrices, underwent a greater change in single-slice analysis (median *B* = 0.36) than in multi-slice analysis (median *B* = 0.13). Fractal features underwent a significant change only in single-slice analysis (median *B* = 0.49).

**Conclusions:**

Incremental levels of ASIR affect significantly CT radiomics quantification in primary colorectal cancer. Second-order statistical and fractal features derived from single-slice analysis undergo greater change than those from multi-slice analysis.

**Key Points:**

*• Incremental levels of ASIR determine a significant change in most statistical (first-, second- and high-order) CT radiomics features measured in primary colorectal cancer, best described by a linear relationship.*

*• First-order statistical features undergo a small change, both from single-slice and multi-slice radiomics analyses.*

*• Most second-order statistical features undergo a greater change in single-slice analysis than in multi-slice analysis. Fractal features are only affected in single-slice analysis.*

## Introduction

There has been a growing interest in radiomics approaches that extract quantitative image features to improve cancer phenotyping [1]. Radiomics have shown promise for tumour characterisation, prognostication, therapy planning and therapy assessment in a number of cancers including non-small cell lung cancer [2–8], breast [9–12], prostate [13, 14] and colorectal cancer [15–21].

Advances in computed tomography (CT) technology, linked to dose reduction, have led to the implementation of iterative image reconstruction algorithms in order to compensate for the increase in image noise with low-dose CT acquisitions [22–26]. Adaptive statistical iterative reconstruction (ASIR) is a hybrid algorithm that uses the image information obtained from filtered back projection (FBP) as the basis for iterative reconstruction to optimise image quality. It has enabled dose reductions between 32 and 65%, without substantially affecting image quality in phantom studies [27, 28].

To date, the majority of published retrospective CT radiomics studies have been based on images reconstructed with FBP alone. Given that iterative reconstruction has been adopted by all manufacturers in current CT scanners, the impact of iterative reconstruction algorithms on quantitative image features is highly relevant to the field of radiomics. We hypothesised that ASIR would alter feature values significantly compared with FBP, and alter features progressively with incremental ASIR weightings (percentage increments). Thus, the primary aim of our study was to investigate whether hybrid iterative reconstruction, specifically ASIR applied with incremental weightings, affects the quantification of radiomics features, including first-, second- and high-order statistical as well as fractal parameters, using primary colorectal cancer at peak contrast enhancement as an exemplar in light of promising data [29]. Our secondary aim was to establish whether features calculated from single-slice analysis (2-dimensional [2D] radiomics) are influenced to a greater degree than those from multi-slice analysis (3-dimensional [3D] radiomics).

## Materials and methods

### Participants

Following institutional review board approval and informed consent, 32 consecutive patients with primary colorectal cancer underwent contrast-enhanced CT from January 2012 to July 2014 from a single institution (prospective trial, ISCTRN 95037515). Exclusion criteria were tumour diameter < 2 cm (to assure a sufficient number of CT voxels for analysis), impaired renal function (estimated glomerular filtration rate < 50 mL/min) and previous iodinated contrast media allergic reaction precluding administration of an iodinated contrast agent.

### CT acquisition and reconstruction

**Fig. 1:**
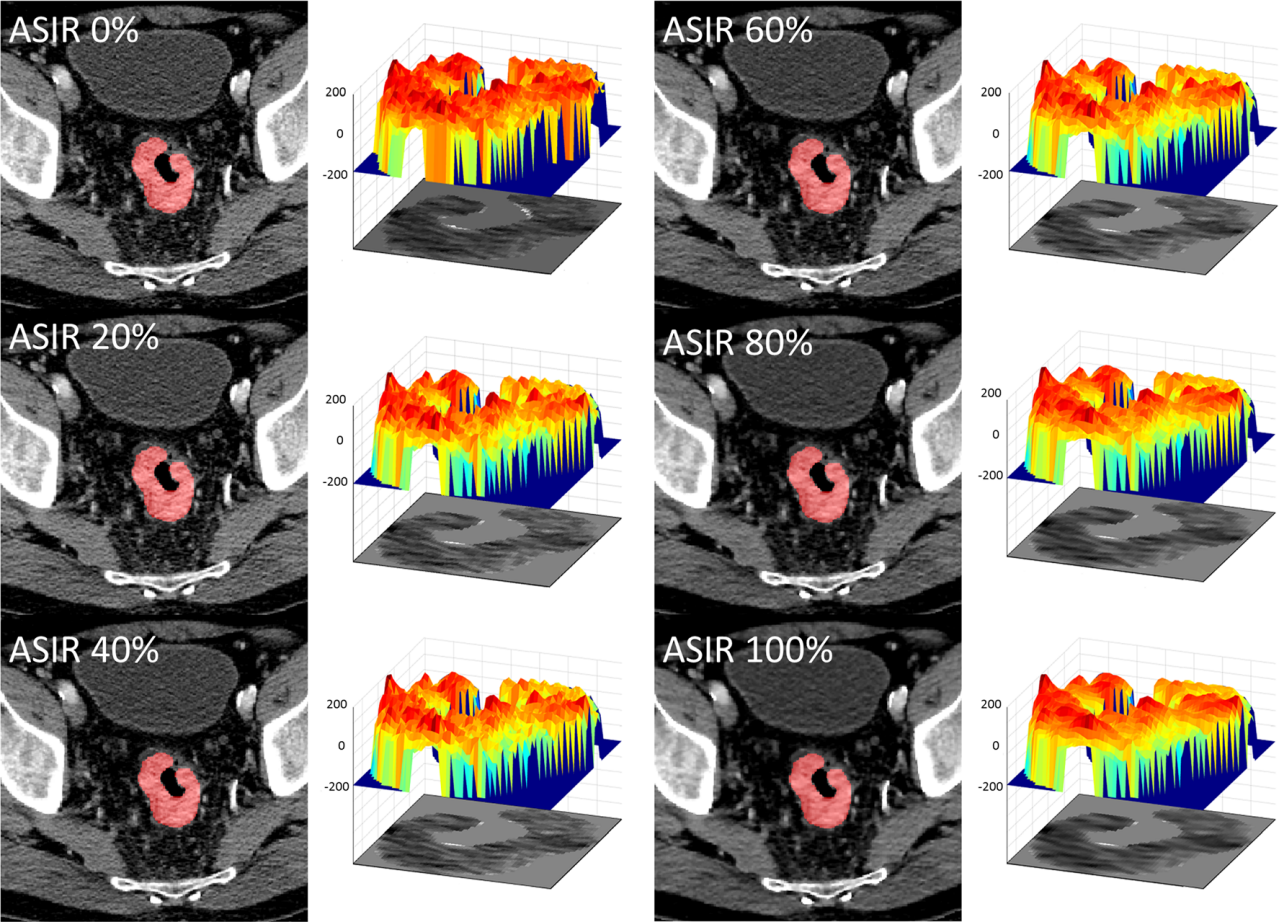
Representative axial CT image reconstructed at 0%, 20%, 40%, 60%, 80% and 100% ASIR, showing a reduction in image noise with increasing ASIR weighting. Corresponding tumour ROI surface histograms, representing voxel values in Hounsfield units (scale − 200 to 200), demonstrate progressive smoothing of the surface as ASIR weighting increases

CT was performed on a single Discovery 750 HD multi-detector CT scanner (GE Healthcare). As part of a prospective research protocol, a dynamic contrast-enhanced CT centred on the primary cancer was acquired using the following protocol: 100 kV; 75 mAs; *z*-axis coverage, 4 cm; scan field of view, 50 cm; matrix, 512 × 512 mm; B30 soft reconstruction kernel; 5 mm reconstructed slice thickness; axial mode with 35 acquisition time points at a 1.5-s temporal resolution for 45 s and a 5-s temporal resolution thereafter for 120 s. To minimise bowel peristaltic movement, 20 mg of the spasmolytic agent hyoscine butylbromide (Buscopan; Boehringer Ingelheim) was administered intravenously prior to data acquisition unless contraindicated. The contrast agent was administered as follows: 50 mL of 370 mg/mL iodinated contrast agent (Niopam, Bracco) via a pump injector (Medrad Stellant dual syringe, Bayer Healthcare) at a rate of 5 mL/s, followed by a 50 ml saline chaser at the same rate. Mean CTDI_Vol_ and DLP were 137.8 ± 15.3 mGy and 551.0 ± 61.2 mGy cm, respectively. The dynamic acquisition was reconstructed at the scanner with six different ASIR percentages: 0%, (equivalent to FBP) 20%, 40%, 60%, 80% and 100%, resulting in six separate datasets per patient (Fig. 1**)**.

### Image analysis

Image analysis was carried out by two readers in consensus (a clinical oncologist and a radiologist with 5 and 10 years CT experience, respectively) using the CT acquisition corresponding to peak tumour enhancement, in order to maximise the tumour contrast-to-noise ratio. A free-hand region of interest (ROI) was drawn around the tumour on 0% ASIR reconstructions to generate two datasets per patient: (1) a single axial image corresponding to the largest tumour area; (2) five contiguous axial images including tumour. ROIs were then copied onto 20–100% ASIR reconstructions to ensure these were identical.

Radiomics features were extracted using an in-house software based on Matlab (Mathworks), previously validated on a digital phantom developed as part of the Image Biomarker Standardisation Initiative [30]. A medium smoothing filter and 32 bin width were applied to the native DICOM images.

First-order (histogram), second-order (grey-level co-occurrence matrix, GLCM; grey-level difference matrix, GLDM) and high-order (neighbourhood grey-tone difference matrix, NGTDM; grey-level run length, GLRL; grey-level zone-size matrix, GLZSM) statistical, as well as model-based fractal features were extracted. The extracted features are summarised in Table 1.

**Table 1:** Summary and brief description of the CT radiomics features extracted

Feature type	Method	Parameters
First-order	Describe the histogram distribution of voxel signal intensity values without spatial information	Mean, maximum, minimum, range, standard deviation, coefficient of variation, skewness, kurtosis, energy, entropy
Second-order grey-level co-occurrence matrix (GLCM)	Describe the statistical interrelationships between grey-level pairs with similar or dissimilar signal intensity values within an imaging plane	Autocorrelation, cluster prominence, cluster shade, contrast, correlation, difference entropy, difference variance, dissimilarity, entropy, energy, homogeneity, information measure correlation, inverse difference moment normalised, inverse difference normalised, maximum probability, sum average, sum entropy, sum of squares variance
Second-order grey-level difference matrix (GLDM)	Describe the grey-level differences of all possible pairs of grey-level distance (d) apart at angle (*Θ)*	Mean, entropy, variance, contrast
High-order neighbourhood grey-tone difference matrix (NGTDM)	Describe the signal intensity and spatial interrelationship between each voxel with its direct 26 neighbours	Coarseness, contrast, busyness, complexity, texture strength
High-order grey-level run length (GLRL)	Describe the spatial interrelationship between neighbouring runs of voxels with the same intensity	Run percentage, high grey-level run emphasis, short-run low grey-level emphasis, short-run high grey-level emphasis, short-run emphasis, long-run emphasis, grey-level non-uniformity, run length non-uniformity, low grey-level run emphasis, long-run low grey-level run emphasis, long-run high grey-level run emphasis, intensity variability, run length variability
High-order grey-level zone-size matrix (GLZSM)	Describe signal intensity and spatial interrelationship between neighbouring zones with the same intensity	Short-zone emphasis, short-zone low-intensity emphasis, short-zone high-intensity emphasis, long-zone low-intensity emphasis, long-zone high-intensity emphasis, long-zone emphasis, intensity non-uniformity
Model-based fractal features	Describe repetitive patterns within an image extracted by using filter grids	Fractal dimension mean, fractal dimension SD, fractal lacunarity, Hurst exponent, blanket mean, blanket max

### Statistical analysis

All radiomics features were regarded as continuous in nature. To allow for the fact that measurements from the same subject were likely to be more similar than from different subjects, the analysis was performed using multilevel linear regression. Two-level models were used, with individual measurements nested within patients. The shape of the relationship between ASIR levels and radiomics parameter values was examined. Initially, cubic, squared and linear terms for ASIR were included in the analysis. If the higher order terms (i.e. cubic and squared terms) were not found to be statistically significant, they were omitted and a simple linear relationship between variables was assumed. The distributions of the majority of radiomics parameters were such that the assumptions of the statistical methods were met. However, one parameter (fractal dimension lacunarity) had a particularly positively skewed distribution, and was thus analysed on the log scale. Regression coefficients along with corresponding confidence intervals were reported, representing the change in the CT parameter value for a 20% increase in ASIR. Standardised effect sizes (*B*) were calculated by the absolute regression coefficient divided by the between-subject standard deviation for a 20-unit increase in ASIR. Conventionally, *B* values ≤ 0.3 are considered representative of a small effect; *B* values > 0.3 and < 0.8 are considered a moderate effect; ≥ 0.8, a large effect. All analyses were undertaken by a statistician using Stata, version 13.1 (StataCorp LP). A *p* value < 0.05 was taken to represent statistical significance.

## Results

### Participants

No tumour was identified on CT in 1 participant (confirmed histological diagnosis of colorectal adenocarcinoma based on a resected polyp). Voxel spatial mismatch between 0% ASIR and subsequent ASIR reconstructed series precluded the analysis in 3 further participants. Therefore, 28 CT datasets were suitable for single-slice (2D) radiomics analysis. Multi-slice (3D) analysis was only possible in a subset of 23 participants, as 5 tumours were not sufficiently large or appropriately oriented to allow segmentation on at least 5 consecutive axial slices. Tumours had a mean diameter of 5.7 ± 1.74 cm and were located as follows: caecum, *n* = 4; ascending colon, *n* = 2; sigmoid colon, *n* = 7; rectum, *n* = 15. Radiological tumour (T) and nodal (N) staging, evaluated on CT images and based on the AJCC/UICC TNM classification of malignant tumours (8th edition), was as follows: T2, *n* = 9; T3, *n* = 17; T4, n = 2; N0, *n* = 16; N1, *n* = 10; N2, *n* = 2.

### Radiomics analysis

Absolute regression coefficients, standardised effect size *B* values and corresponding *p* values for first-order, second-order and fractal radiomics features from single-slice and multi-slice datasets are summarised in Table 2; values for high-order features from multi-slice datasets are summarised in Table 3.

**Table 2:** Summary of results for first-order, second-order and fractal features from single-slice (2D) and multi-slice (3D) analysis. For each radiomics feature, the regression coefficients (95% confidence intervals [CI]) and standardised effect size (B) for a 20% increase in ASIR, and the corresponding *p* values, are presented. Significant *p* values are marked in italics

CT radiomics	2D regression coefficient (95% CI)	2D *Β* value*	2D *p* value	3D regression coefficient (95% CI)	3D *Β* value*	3D *p* value
First-order features						
Mean	− 0.05 (− 0.06, − 0.03)	0.01	*< 0.001*	− 0.18 (− 0.30, − 0.05)	0.01	*0.005*
Maximum	− 5.9 (− 6.3, − 5.2)	0.24	*< 0.001*	− 6.1 (− 6.8, − 5.3)	0.27	*< 0.001*
Minimum	3.9 (3.0, 4.8)	0.04	*< 0.001*	− 8.0 (− 13.6, − 2.5)	0.04	*0.005*
Range	− 9.8 (− 11.2, − 8.3)	0.09	*< 0.001*	2.0 (− 3.6, 7.6)	0.01	*0.48*
Standard deviation	− 1.27 (− 1.44, − 1.10)	0.18	*< 0.001*	− 0.78 (− 1.04, − 0.52)	0.06	*< 0.001*
Coefficient of variation	− 0.022 (− 0.025, − 0.018)	0.10	*< 0.001*	− 0.010 (− 0.019, − 0.002)	0.02	*0.02*
Kurtosis	0.33 (0.21, 0.45)	0.05	*< 0.001*	2.1 (1.4, 2.8)	0.10	*< 0.001*
Skewness	− 0.064 (− 0.078, − 0.052)	0.08	*< 0.001*	− 0.18 (− 0.23, − 0.13)	0.12	*< 0.001*
Energy	0.0010 (0.0007, 0.0015)	0.04	*< 0.001*	0.005 (0.004, 0.007)	0.11	*< 0.001*
Entropy	− 0.008 (− 0.013, − 0.003)	0.02	*0.003*	− 0.047 (− 0.060, − 0.035)	0.10	*< 0.001*
Second-order features						
GLCM Autocorrelation	8.5 (7.2, 9.9)	0.19	*< 0.001*	68 (54, 82)	0.11	*< 0.001*
GLCM Cluster prominence	787 (555, 1018)	0.07	*< 0.001*	− 70 (− 92, − 48)	0.10	*< 0.001*
GLCM cluster shade	− 28 (− 35, − 20)	0.12	*< 0.001*	4147 (2692, 5602)	0.09	*< 0.001*
GLCM contrast	− 2.9 (− 3.2, − 2.5)	0.35	*< 0.001*	− 2.4 (− 3.0, − 1.8)	0.10	*< 0.001*
GLCM correlation	0.038 (0.034, 0.042)	0.50	*< 0.001*	0.017 (0.014, 0.020)	0.31	*< 0.001*
GLCM difference entropy	− 0.053 (− 0.060, − 0.048)	0.44	*< 0.001*	− 0.05 (− 0.06, − 0.04)	0.16	*< 0.001*
GLCM difference variance	− 2.9 (− 3.2, − 2.5)	0.35	*< 0.001*	− 2.4 (− 3.0, − 1.8)	0.10	*< 0.001*
GLCM dissimilarity	− 0.23 (− 0.26, − 0.20)	0.38	*< 0.001*	− 0.20 (− 0.24, − 0.16)	0.14	*< 0.001*
GLCM entropy	− 0.056 (− 0.063, − 0.050)	0.37	*< 0.001*	− 0.09 (− 0.11, − 0.07)	0.13	*< 0.001*
GLCM energy	0.0003 (0.0003, 0.0004)	0.30	*< 0.001*	0.0010 (0.0008, 0.0012)	0.16	*< 0.001*
GLCM homogeneity	0.012 (0.011, 0.014)	0.43	*< 0.001*	0.013 (0.011, 0.015)	0.19	*< 0.001*
GLCM Information measure correlation 1	− 0.010 (− 0.012, − 0.009)	0.24	*< 0.001*	− 0.008 (− 0.010, − 0.007)	0.35	*< 0.001*
GLCM information measure correlation 2	0.017 (0.014, 0.019)	0.16	*< 0.001*	0.013 (0.010, 0.015)	0.15	*< 0.001*
GLCM Inverse difference moment normalised	0.0024 (0.0021, 0.0027)	0.36	*< 0.001*	0.0006 (0.0004, 0.0007)	0.11	*< 0.001*
GLCM inverse difference normalised	0.0052 (0.0046, 0.0057)	0.39	*< 0.001*	0.0027 (0.0022, 0.0032)	0.14	*< 0.001*
GLCM maximum probability	0.0009 (0.0007, 0.0010)	0.40	*< 0.001*	0.0024 (0.0019, 0.0029)	0.17	*< 0.001*
GLCM sum average	0.40 (0.33, 0.47)	0.17	*< 0.001*	1.40 (1.11, 1.68)	0.11	*< 0.001*
GLCM sum entropy	0.000 (− 0.001, 0.002)	0.01	0.76	− 0.026 (− 0.035, − 0.018)	0.07	*< 0.001*
GLCM Sum of squares variance	7.1 (5.9, 8.3)	0.17	*< 0.001*	67 (53, 81)	0.11	*< 0.001*
GLDM mean	− 0.23 (− 0.26, − 0.20)	0.38	*< 0.001*	− 0.20 (− 0.24, − 0.16)	0.14	*< 0.001*
GLDM entropy	− 0.054 (− 0.060, − 0.048)	0.44	*< 0.001*	− 0.05 (− 0.06, − 0.04)	0.16	*< 0.001*
GLDM variance	− 0.96 (− 1.08, − 0.84)	0.36	*< 0.001*	− 0.64 (− 0.83, − 0.44)	0.08	*< 0.001*
GLDM contrast	− 2.86 (− 3.21, − 2.50)	0.35	*< 0.001*	− 2.38 (− 2.96, − 1.81)	0.10	*< 0.001*
Fractal features						
Fractal lacunarity ^+^	0.09 (0.08, 0.10)	0.55	*< 0.001*	–	–	0.12
Fractal dimension mean	− 0.029 (− 0.033, − 0.026)	0.82	*< 0.001*	–	–	0.69
Fractal dimension SD	0.011 (0.010, 0.013)	0.43	*< 0.001*	–	–	0.77
Hurst exponent	0.029 (0.026, 0.033)	0.82	*< 0.001*	–	–	0.69
Blanket mean	− 0.056 (− 0.062, − 0.050)	0.25	*< 0.001*	–	–	0.41
Blanket max	− 0.056 (− 0.062, − 0.050)	0.25	*< 0.001*	–	–	0.41

*Calculated as absolute regression coefficient divided by between-subject standard deviation; ^+^ Variable analysed on the log scale

**Table 3:** Summary of results for high-order features obtained from multi-slice (3D) analysis. For each texture feature, the regression coefficients (95% confidence intervals [CI]) and standardised effect size (B) for a 20% increase in ASIR, and the corresponding *p* values, are presented. Significant *p* values are marked in italics

CT radiomics	Regression coefficient (95% CI)	*Β* value*	*p* value
High-order features			
NGTDM coarseness	− 0.07 (− 0.09, − 0.05)	0.05	*< 0.001*
NGTDM contrast	− 0.004 (− 0.006, − 0.002)	0.04	*0.001*
NGTDM busyness	0.025 (0.016, 0.034)	0.04	*< 0.001*
NGTDM complexity	–	–	0.16
NGTDM texture strength	0.039 (0.024, 0.054)	0.08	*< 0.001*
GLRL run percentage	− 0.010 (− 0.012, − 0.009)	0.22	*< 0.001*
GLRL high grey-level run emphasis	0.25 (0.17, 0.32)	0.01	*< 0.001*
GLRL short-run low grey-level emphasis ^#^	–	–	0.85
GLRL short-run high grey-level emphasis	0.16 (0.10, 0.22)	0.006	*< 0.001*
GLRL short-run emphasis	0.0004 (0.0003, 0.0005)	0.02	*< 0.001*
GLRL long-run emphasis	48 (37, 58)	0.11	*< 0.001*
GLRL grey-level non-uniformity	− 209 (− 273, − 146)	0.06	*< 0.001*
GLRL run length non-uniformity	–	–	0.27
GLRL low grey-level run emphasis	− 0.009 (− 0.010, − 0.007)	0.22	*< 0.001*
GLRL long-run low grey-level emphasis	18.9 (11.1, 26.7)	0.06	*< 0.001*
GLRL long-run high grey-level emphasis	344 (279, 410)	0.24	*< 0.001*
GLRL intensity variability	− 37,623 (− 56,176; − 19,070)	0.05	*< 0.001*
GLRL run length variability	–	–	0.53
GLZLSM short-zone emphasis ^+^	0.05 (0.04, 0.06)	0.17	*< 0.001*
GLZLSM short-zone low-intensity emphasis ^#^	5.3 (2.9, 7.7)	0.13	*< 0.001*
GLZLSM short-zone high-intensity emphasis	0.048 (0.033, 0.063)	0.17	*< 0.001*
GLZLSM long-zone low-intensity emphasis	− 9.8 (− 16.4, − 3.2)	0.04	*0.004*
GLZLSM long-zone high-intensity emphasis	544,100 (368,325; 719,875)	0.18	*< 0.001*
GLZLSM long-zone emphasis	47 (35, 59)	0.09	*< 0.001*
GLZLSM intensity non-uniformity	− 145 (− 190, − 102)	0.14	*< 0.001*
GLZLSM zone length non-uniformity	− 11.5 (− 15.3, − 7.7)	0.10	*< 0.001*
GLZLSM zone percentage	− 0.25 (− 0.028, − 0.021)	0.20	*< 0.001*
GLZLSM low-intensity zone emphasis	− 0.015 (− 0.017, − 0.013)	0.31	*< 0.001*
GLZLSM high-intensity zone emphasis	161 (110, 212)	0.17	*< 0.001*
GLZLSM intensity variability	− 3503 (− 5140, − 1866)	0.14	*< 0.001*
GLZLSM size zone variability	− 339 (− 511, − 167)	0.13	*< 0.001*

* Calculated as absolute regression coefficient divided by between-subject standard deviation; ^+^ variable analysed on the log scale. ^#^ Figures reported in units of 10^−5^

#### Single-slice analysis

All first-order, second-order and fractal features varied significantly and according to a linear relationship with increasing ASIR values, with the exception of GLCM sum entropy (Table 2). Some features significantly increased; others decreased.

The relative effect was small on all first-order features (median standardised effect size *B* = 0.08; range, 0.02–0.24). Most second-order features, including all grey-level difference matrix (GLDM) and the majority of grey-level co-occurrence matrix (GLCM) features, were moderately affected (median *B* = 0.36; range, 0.01–0.44). The relative effect on most fractal features was moderate to large (median *B* = 0.49; range, 0.25–0.82).

#### Multi-slice analysis

Nearly all first-order, second-order and high-order features varied significantly with increasing ASIR values: some increasing, others decreasing. Exceptions were first-order range, neighbourhood grey-tone difference matrix (NGTDM) complexity and 3 out of 13 grey-level run length (GLRL) features (Tables 2 and 3).

A small effect was confirmed on first-order statistical features (median *B* = 0.08; range, 0.01–0.27). Second-order features were affected to a lesser degree than from single-slice analysis (median *B* = 0.13; range, 0.07–0.35): 21 out of 23 features were affected in small measure, compared to 9 out of 23 from single-slice analysis. ASIR effect was small on high-order features (median *B* = 0.09; range, 0.01–0.31). In contrast to single-slice analysis and to all other features, multi-slice fractal features did not change significantly with increasing ASIR levels.

## Discussion

Despite the rising number of studies investigating the clinical potential of radiomics in cancer imaging, relatively little is known on how the shift of CT image reconstruction from filtered back projection to hybrid iterative algorithms might affect radiomics features quantitation. In our prospective study, we found that the application of incremental ASIR levels altered most statistical (first-, second- and high-order) features according to a linear relationship, both in single-slice and multi-slice analysis. Fractal features changed significantly only in single-slice datasets. While first-order features underwent small relative effects across all datasets, second-order features underwent a greater change in single-slice than in multi-slice analysis.

A fundamental aspect of CT is the assignment of an attenuation value to a voxel. Analytical reconstruction algorithms, namely filtered back projection, have long been the backbone of CT reconstruction. However, as image noise from the Poisson statistical variation across an image is not accounted for by FBP; this has been a limiting factor for low-dose CT imaging [31]. Iterative reconstruction algorithms have thus risen to the fore with advances in scanner hardware and increasing computing power [31]. Hybrid algorithms such as ASIR combine both analytical and iterative methods, optimising image characteristics by decreasing image noise. However, there is a perceptive alteration in image characteristics with high ASIR increments often described as a ‘waxy’ appearance. Given this, we hypothesised that radiomics features may be altered by using ASIR compared to FBP and the ASIR weighting.

To date, only one other clinical study has compared the effect of different reconstruction algorithms on quantitative CT data including a limited number of histogram and second-order GLCM features. The effect of FBP, 50% ASIR and model-based iterative reconstruction (MBIR) was compared for volumes of interest in 20 patients with liver lesions (*n* = 13), lung nodules (*n* = 9) or renal calculi (*n* = 25) who underwent either non-enhanced CT or contrast-enhanced CT at a fixed 120 kVp but 2 different dose levels (full and half-dose) [32]. MBIR had the highest impact on features. Fifty percent ASIR had a significant effect on standard deviation (SD) but not on other first-order (entropy, kurtosis or skewness) or second-order GLCM features studied. These differences in part reflect a difference in study design, i.e. assessment of an incremental effect of ASIR as opposed to a comparison of a single ASIR percentage to FBP and MBIR.

We acknowledge that the reconstruction algorithm is only one of several factors that potentially affect CT radiomics features. Other factors include acquisition factors, e.g. kVp, mAs, reconstruction kernel, voxel size, grey-level discretisation and contrast administration [32–39]. For example, Zhao et al investigated the effect of reconstruction kernel on 89 unenhanced CT radiomics features including shape, first-order, second-order statistical, wavelet and fractal features for 32 lung cancers using 2D and 3D images. The reconstruction kernel had a significant effect on extracted features, with smooth images having a smaller effect than noisier images. 3D images were also more reproducible than 2D images [34]. This was also echoed in our study where 3D features were more stable.

He et al assessed the effects of reconstruction slice thickness, reconstruction kernel and contrast-enhancement on the diagnostic performance of 150 radiomics features in 240 patients with solitary pulmonary nodules (malignant, *n* = 180; benign, *n* = 60). This study demonstrated better discrimination and classification for malignant versus benign nodules when based on unenhanced versus contrast-enhanced CT, thin- (1.25 mm) versus thick-slice CT (5 mm) and standard versus lung reconstruction kernel [35]. In a further study, Shafiq-Ul-Hassan et al found that voxel size and discretisation were also important factors affecting radiomics features including shape, intensity, GLCM, GLZSM, GLRL, NGTDM, fractal and wavelet features in a digital phantom [39].

From our study and published data, minimisation of variation in reconstruction kernel, reconstruction algorithm, voxel size and grey-level discretisation would improve the quantification and stability of CT radiomics features.

We acknowledge some limitations to our study. Firstly, the study cohort was small (*n* = 28) but with the advantage of fixed acquisition parameters, including kVp and mAs, reconstruction kernel, and voxel size, allowing us to focus on the effect of the reconstruction algorithm alone. Secondly, we did not assess the effect of other iterative reconstruction algorithms as these were not available to us at the time of acquisition; we acknowledge that this would be of value going forward in future studies.

In conclusion, we confirmed that the application of a hybrid reconstruction algorithm versus traditional FBP affects CT radiomics quantification. The use of multi-slice (3D) rather than single-slice (2D) data will minimise this effect, particularly for second-order statistical and model-based fractal features. The reconstruction algorithm should be taken into account and standardised when acquiring data for future multicentre CT radiomics studies.

## Abbreviations

2D: 2-dimensional
3D: 3-dimensional
ASIR: Adaptive statistical iterative reconstruction
CT: Computed tomography
FBP: Filtered back projection
GLCM: Grey-level co-occurrence matrix
GLDM: Grey-level difference matrix
GLRL: Grey-level run length
GLZSM: Grey-level zone-size matrix
MBIR: Model-based iterative reconstruction
NGTDM: Neighborhood grey-tone difference matrix
ROI: Region of interest
SD: Standard deviation
